# Supplementary material for: Refining the identity of mesenchymal cell types associated with murine periosteal and endosteal bone
Source: J Biol Chem. 2024 Mar 11;300(4):107158. doi: 10.1016/j.jbc.2024.107158 (PMC11007436; doi:10.1016/j.jbc.2024.107158)
Supplement: Supporting Information [file mmc1.pdf]

Refining the identity of mesenchymal cell types associated with murine periosteal and endosteal bone

Intawat Nookaew, Jinhu Xiong, Melda Onal, Cecile Bustamante-Gomez, Visanu Wanchai, Qiang Fu, Ha-Neui Kim, Maria Almeida, and Charles A. O’Brien

Figures S1-S8

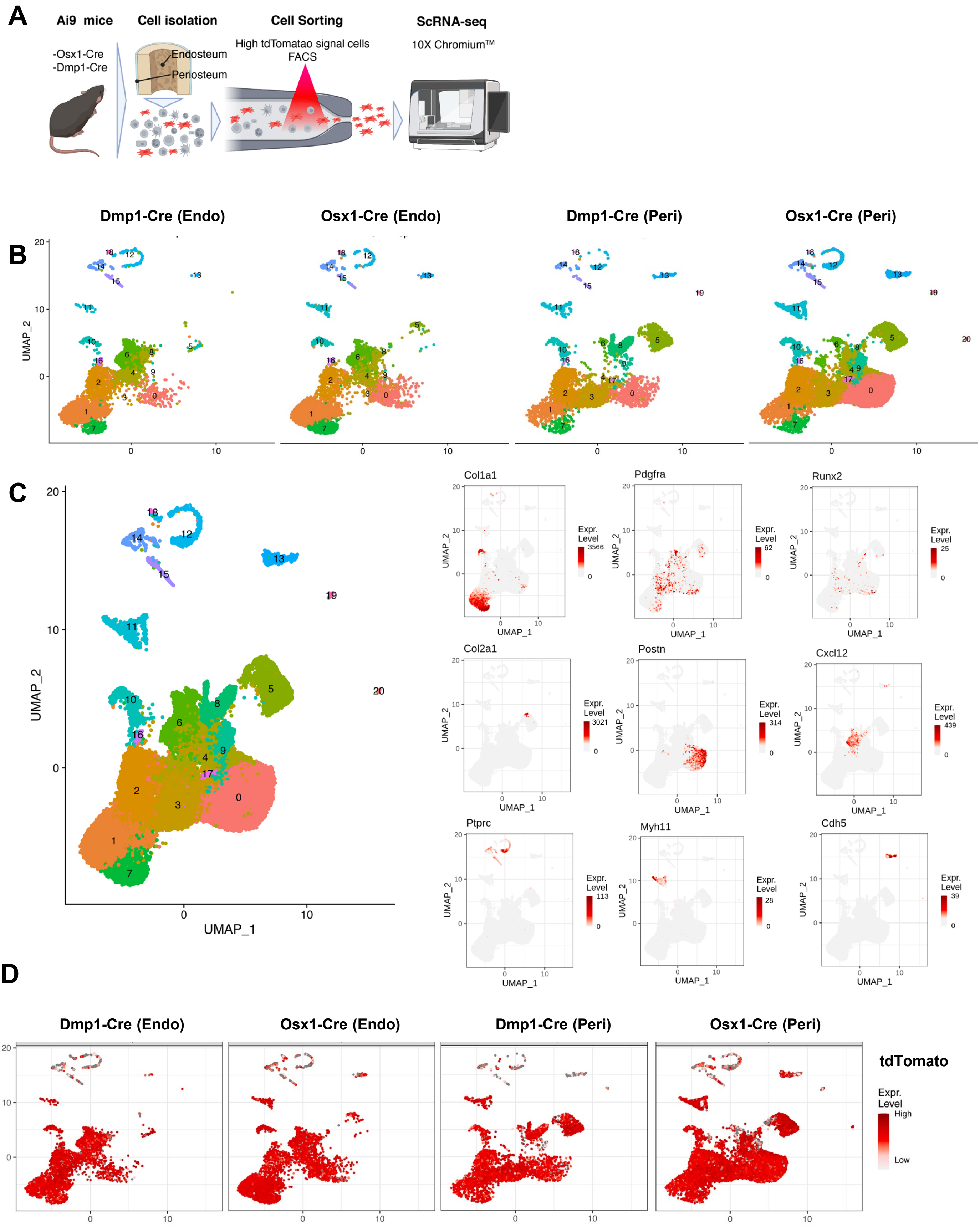
A
Ai9 mice
Cell isolation
Cell Sorting
ScRNA-seq
-Osx1-Cre
-Dmp1-Cre
Endosteum
Periosteum
High tdTomatao signal cells
FACS
10X Chromium™
B
Dmp1-Cre (Endo)
Osx1-Cre (Endo)
Dmp1-Cre (Peri)
Osx1-Cre (Peri)
UMAP_2
C
UMAP_1
UMAP_2
Col1a1
Pdgfra
Runx2
Col2a1
Postn
Cxcl12
Ptprc
Myh11
Cdh5
Expr. Level
D
Dmp1-Cre (Endo)
Osx1-Cre (Endo)
Dmp1-Cre (Peri)
Osx1-Cre (Peri)
tdTomato
Expr. Level
High
Low
UMAP_2

Figure S1

Figure S1. Initial cluster analysis of Dmp1-Cre and Osx1-Cre targeted cells. A. Diagram of the approach used to perform single-cell RNA-seq of cells from Dmp1-Cre;Ai9 and Osx1-Cre;Ai9 mice in this study. **B.** Initial UMAP representations of cell clusters (0-20) targeted by Dmp1-Cre or Osx1-Cre in endosteal and trabecular bone (Endo) or in periosteal bone (Peri). The Dmp1-Cre endosteal preparation represents 3,845 cells derived form the femurs and tibias of 2 mice. The Osx1-Cre endosteal preparation represent 6,071 cells derived from the femurs and tibias of 1 mouse. The Dmp1-Cre periosteal preparation represents 4,106 cells derived from the femurs of 2 mice. The Osx1-Cre periosteal preparation represents 15,113 cells derived from the femurs of 4 mice. **C.** The left panel is a combined UMAP of all the cells in panel B with color-coded clusters (0-20) and the UMAP feature plots on the right show expression of the indicated transcripts. Red = high expression. **D.** UMAP feature plots showing levels of the tandem-dimer tomato mRNA, produced by the activated reporter gene in Ai9 mice, in each of the cell preparations shown in panel A. Red = high expression.

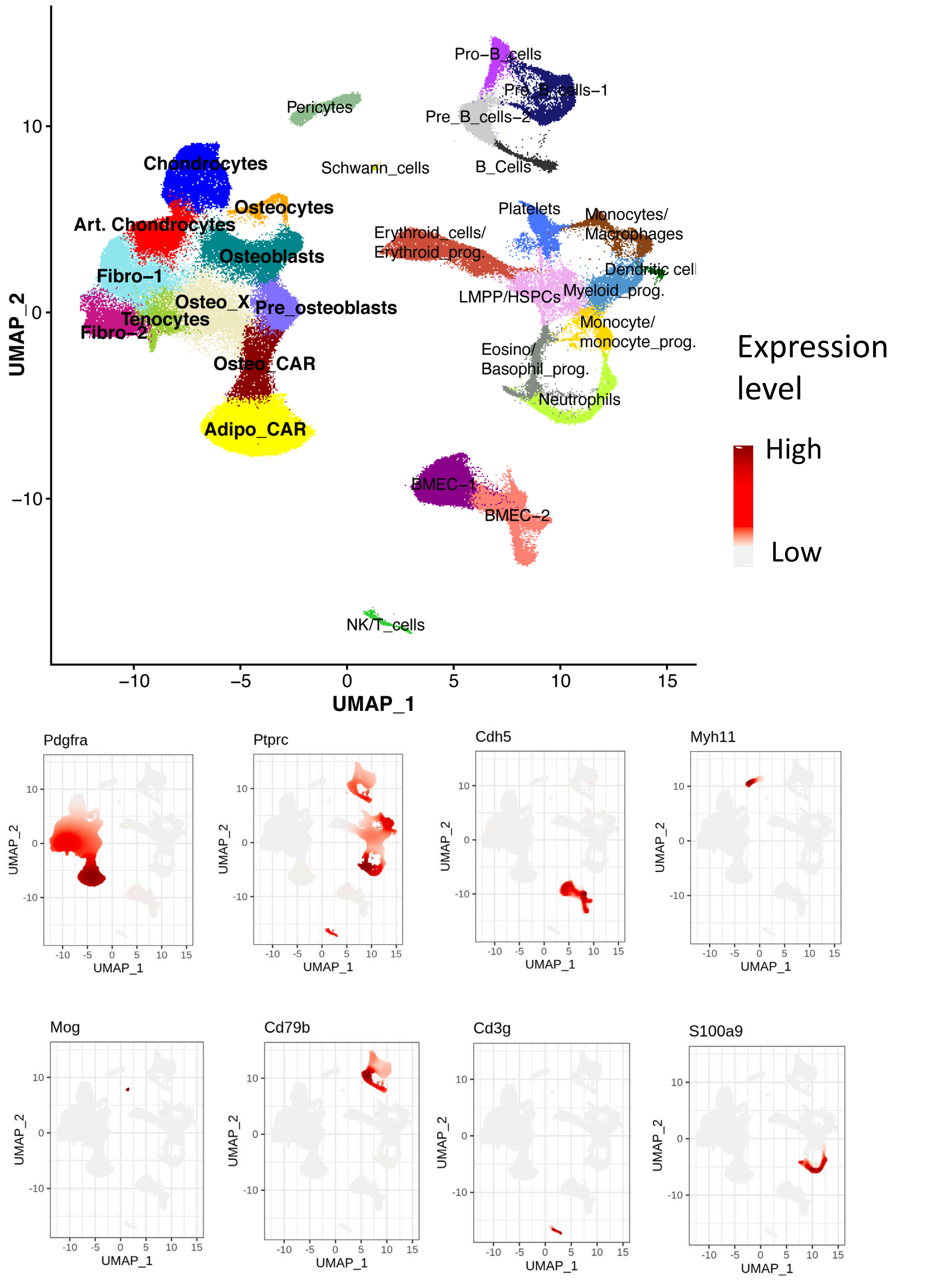

Pro−B_cells
Pre_B_cells−1
Pre_B_cells−2
B_Cells
Pericytes
Schwann_cells
Chondrocytes
Osteocytes
Art. Chondrocytes
Osteoblasts
Fibro−1
Osteo_X
Pre_osteoblasts
Tenocytes
Fibro−2
Osteo_CAR
Adipo_CAR
Platelets
Monocytes/
Macrophages
Erythroid_cells/
Erythroid_prog.
Dendritic cell
LMPP/HSPCs
Myeloid_prog.
Monocyte/
monocyte_prog.
Eosino/
Basophil_prog.
Neutrophils
BMEC−1
BMEC−2
NK/T_cells
UMAP_2
UMAP_1
Expression
level
High
Low
Pdgfra
Ptprc
Cdh5
Myh11
Mog
Cd79b
Cd3g
S100a9

Figure S2

Figure S2. Cluster analysis of combined datasets. The top panel is a UMAP representation all cell clusters identified in the combined dataset consisting of the cells isolated from Dmp1-Cre and Osx1-Cre mice in this study as well as cells isolated in 9 published studies. The bottom panels are UMAP feature plots showing expression of the indicated genes that are characteristic of specific cell types: *Ptprc* (encoding Cd45) – hematopoietic cells; *Pdgfra* – mesenchymal cells; *Cdh5* – endothelial cells; *Myh11* – pericytes; *Mog* – Schwann cells; *Cd79b* – B lymphocytes; *Cd3g* – T lymphocytes; *S100a9* – myeloid cells. Red = high expression.

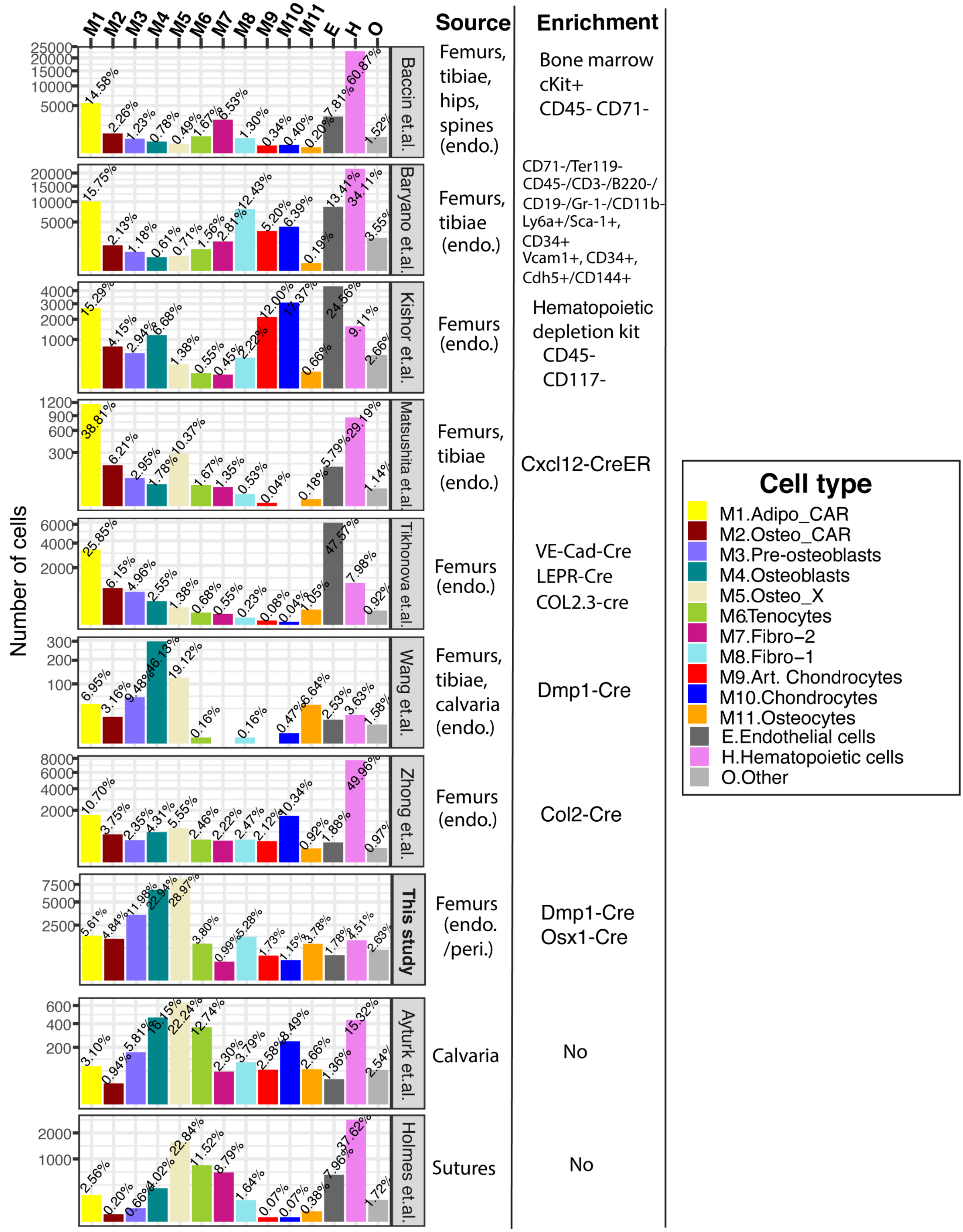

M1
M2
M3
M4
M5
M6
M7
M8
M9
M10
M11
E
H
O
Number of cells
Source
Enrichment
Baccin et.al.
Femurs, tibiae, hips, spines (endo.)
Bone marrow cKit+ CD45- CD71-
Baryano et.al.
Femurs, tibiae (endo.)
CD71-/Ter119- CD45-/CD3-/B220-/ CD19-/Gr-1-/CD11b- Ly6a+/Sca-1+, CD34+ Vcam1+, CD34+, Cdh5+/CD144+
Kishor et.al.
Femurs (endo.)
Hematopoietic depletion kit CD45- CD117-
Matsushita et.al.
Femurs, tibiae (endo.)
Cxcl12-CreER
Tikhonova et.al.
Femurs (endo.)
VE-Cad-Cre LEPR-Cre COL2.3-cre
Wang et.al.
Femurs, tibiae, calvaria (endo.)
Dmp1-Cre
Zhong et.al.
Femurs (endo.)
Col2-Cre
This study
Femurs (endo. /peri.)
Dmp1-Cre Osx1-Cre
Ayturk et.al.
Calvaria
No
Holmes et.al.
Sutures
No
Cell type
M1.Adipo_CAR
M2.Osteo_CAR
M3.Pre-osteoblasts
M4.Osteoblasts
M5.Osteo_X
M6.Tenocytes
M7.Fibro−2
M8.Fibro−1
M9.Art. Chondrocytes
M10.Chondrocytes
M11.Osteocytes
E.Endothelial cells
H.Hematopoietic cells
O.Other

Figure S3

Figure S3. Comparison of cell yields for different studies. Bar graphs showing the relative and absolute abundance of different cell types isolated in datasets from published studies used here as well as the cells isolated from Dmp1-Cre and Osx1-Cre mice in the current study. (endo) = efforts were made to remove periosteal cells from long bones before digestion with collagenase.

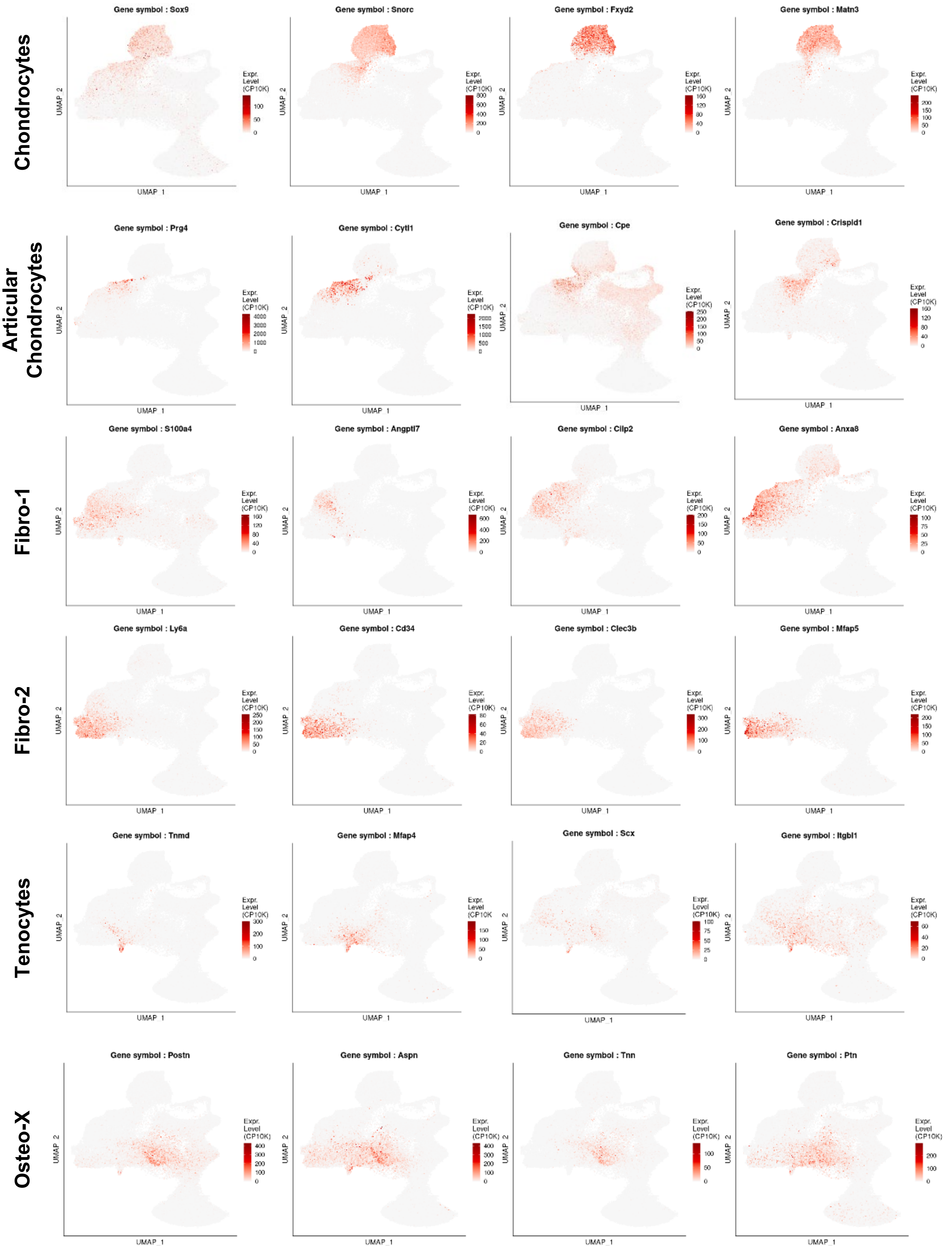
Chondrocytes
Gene symbol : Sox9
Gene symbol : Snorc
Gene symbol : Fxyd2
Gene symbol : Matn3
Articular Chondrocytes
Gene symbol : Prg4
Gene symbol : Cytl1
Gene symbol : Cpe
Gene symbol : Crispld1
Fibro-1
Gene symbol : S100a4
Gene symbol : Angptl7
Gene symbol : Cilp2
Gene symbol : Anxa8
Fibro-2
Gene symbol : Ly6a
Gene symbol : Cd34
Gene symbol : Clec3b
Gene symbol : Mfap5
Tenocytes
Gene symbol : Tnmd
Gene symbol : Mfap4
Gene symbol : Scx
Gene symbol : Itgbl1
Osteo-X
Gene symbol : Postn
Gene symbol : Aspn
Gene symbol : Tnn
Gene symbol : Ptn
UMAP_1
UMAP_2
Expr. Level (CP10K)

Figure S4

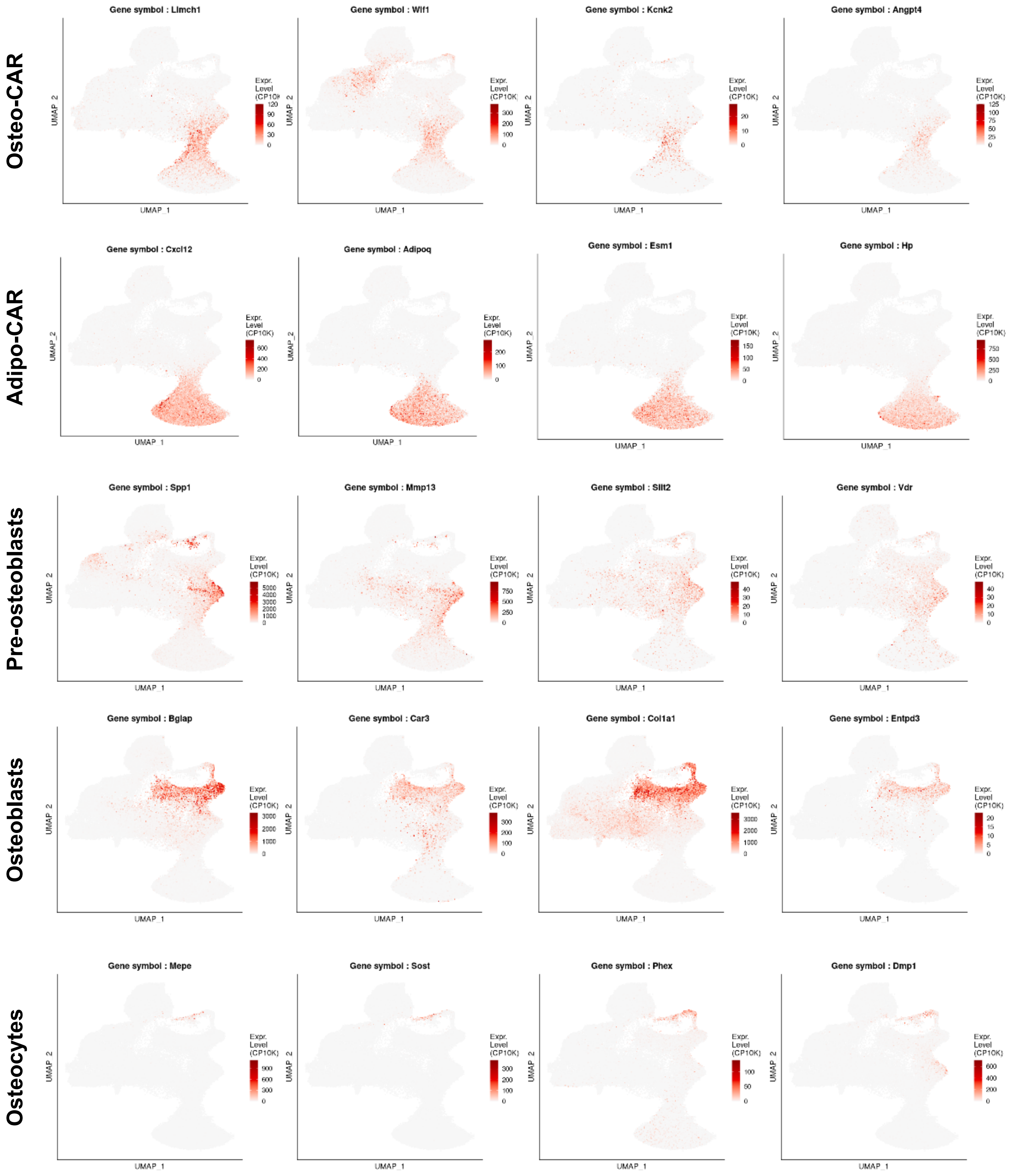

Figure S4. UMAP feature plots of the indicated transcripts in mesenchymal cells of the combined dataset. Red = high expression.

Figure S4 continued

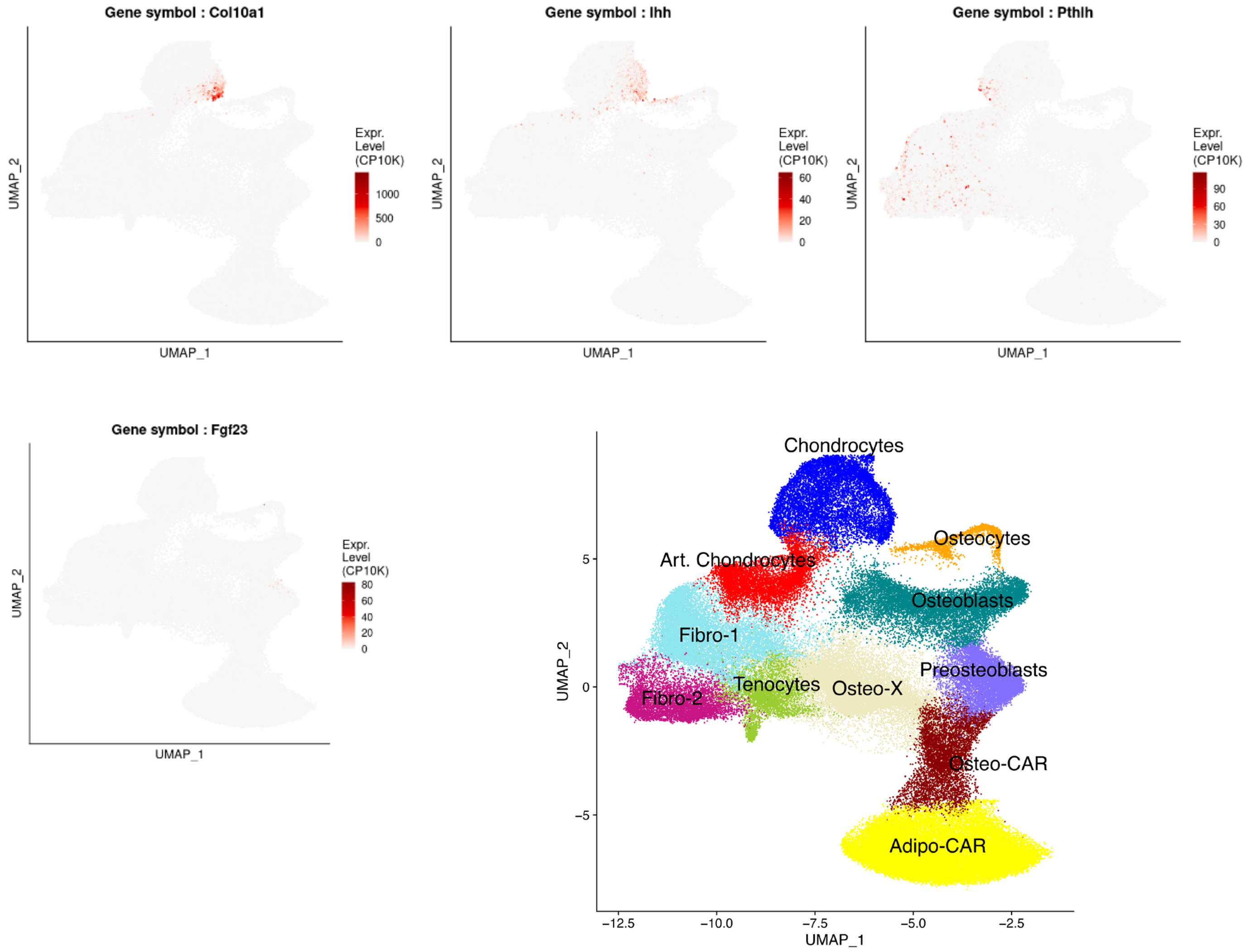

Figure S5. UAMP feature plots of the indicated genes in mesenchymal cells of the combined dataset. Red = high expression. Please note that the UMAP at the bottom right is the same as in main Figure 1B and is presented as a reference to aid in cell type identification.

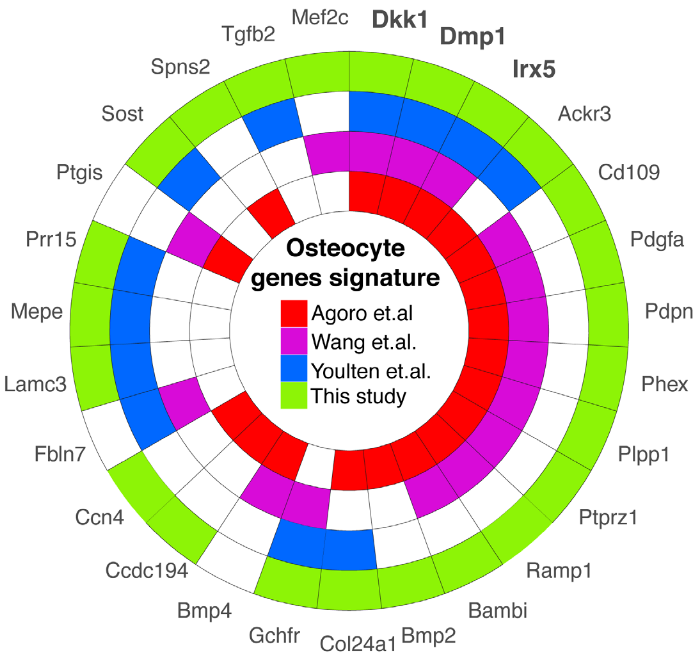

Figure S6. Polar plot of osteocyte marker genes. The top osteocyte marker genes identified from our study were compared with previously reported osteocyte marker genes by others: Agoro et al. (37), Youlten et al. (38), and Wang et al. (26). Only genes that were identified in at least two of the four studies were included in the polar plot.

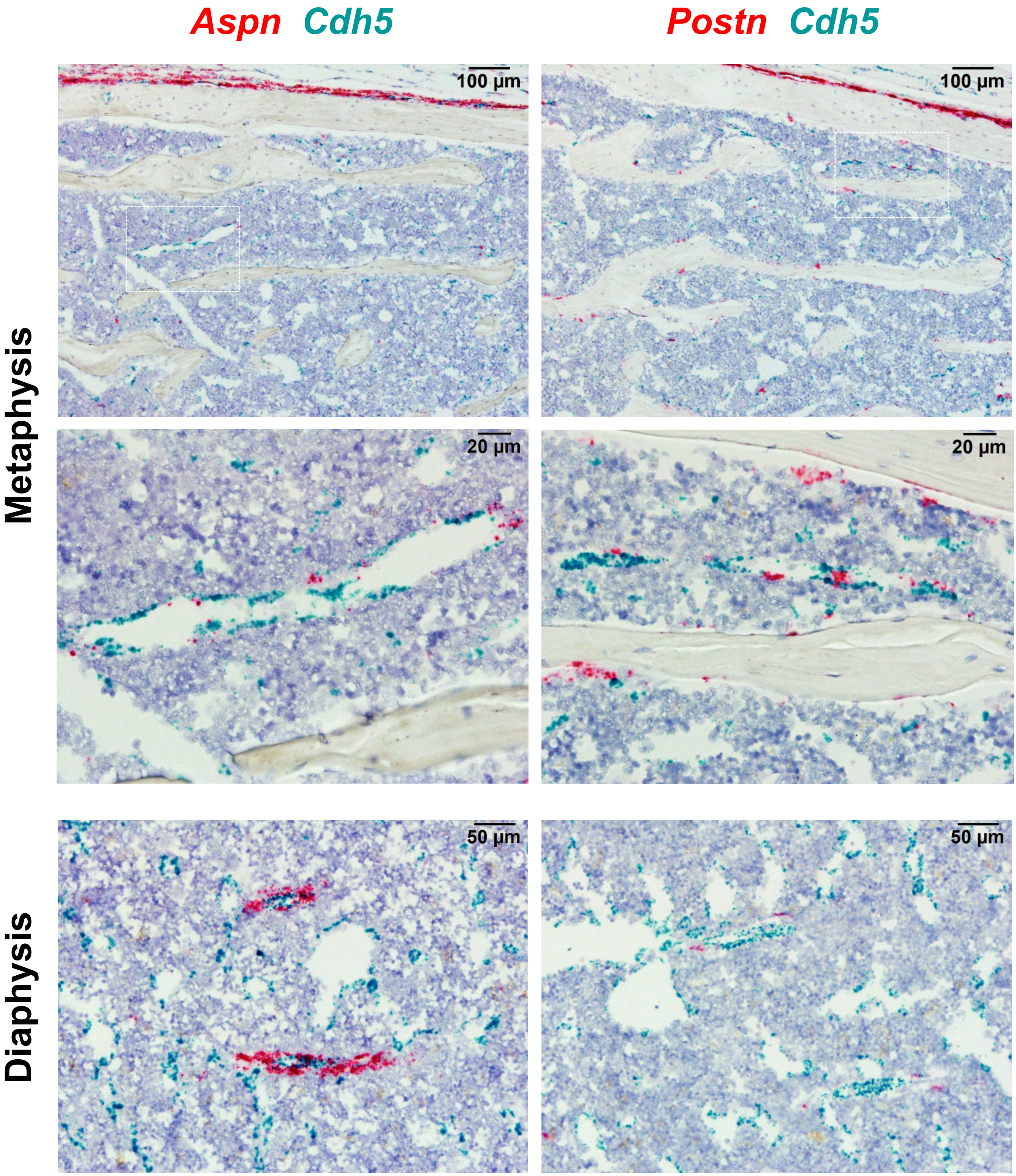

Figure S7. Localization of osteo-X cells and blood vessel endothelial cells. Two-color RNAScope-based in situ hybridization using femoral bone sections from a 4-month-old female C57BL/6 mouse. Probes for *Aspn* and *Postn* (red) are representative of osteo-X cells and the probe for *Cdh5* (teal) is representative of blood vessel endothelial cells. Higher magnification images of metaphyseal regions of the top panels are shown below them.

Figure S7

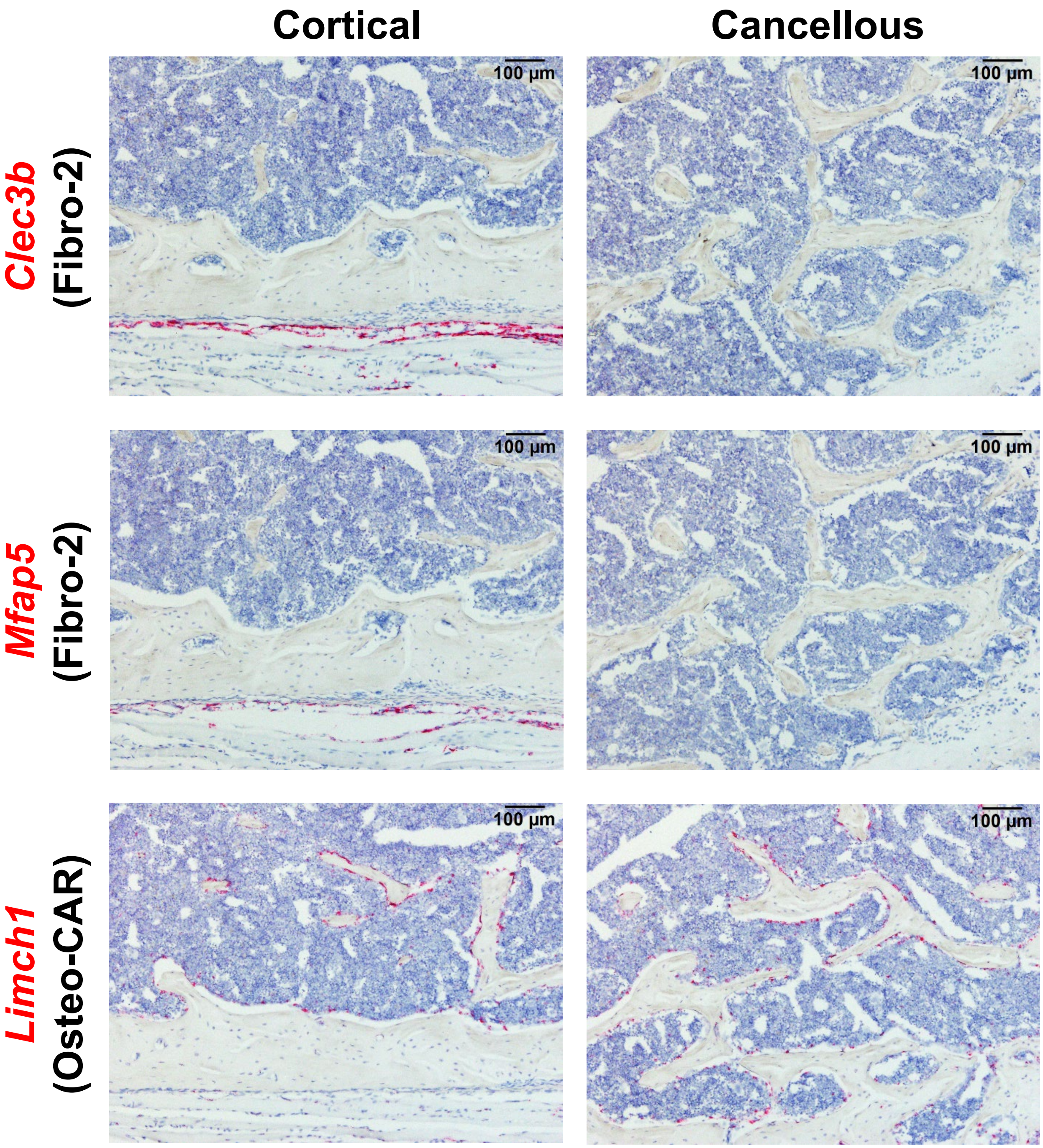

Figure S8. The bone marrow cavity contains few cells with mesenchymal stem cell markers. Single color RNAScope-based in situ hybridization of femoral bone sections from a 4-month-old female C57BL/6 mouse. The identity of the probes is shown at the left. Red = transcript expression.

Figure S8
